# Years of life lost due to lower extremity injury in association with dementia, and care need: a 6-year follow-up population-based study using a multi-state approach among German elderly

**DOI:** 10.1186/s12877-016-0184-7

**Published:** 2016-01-12

**Authors:** Ying Zhou, Hein Putter, Gabriele Doblhammer

**Affiliations:** Institute for Sociology and Demography, University of Rostock, Ulmenstrasse 69, 18057 Rostock, Germany; Rostock Center for the Study of Demographic Change, Konrad-Zuse-Str. 1, 18057 Rostock, Germany; Department of Medical Statistics and Bioinformatics, Leiden University Medical Centre, Einthovenweg 20, 2333 ZC Leiden, Netherlands; German Center for Neurodegenerative Disease, Ludwig-Erhard-Allee 2, 53175 Bonn, Germany

**Keywords:** Long-term care, Mortality, Cohort analysis, Dementia, Multi-state analysis

## Abstract

**Background:**

Dementia and care need are challenging aging populations worldwide. Lower extremity injury (LEI) in the elderly makes matters worse. Using a multi-state approach, we express the effect of LEI on dementia, care need, and mortality in terms of remaining life expectancy at age 75 (rLE) and years of life lost (YLL).

**Methods:**

A population-based random sample of beneficiaries aged 75–95 years was drawn from the largest public health insurer in Germany in 2004 and followed until 2010 (N 62,103; Mean Age ± SD 81.5 ± 4.8 years; Female 71.2 %). We defined a five-state model (*Healthy*, *Dementia*, *Care*, *Dementia & Care*, *Dead*), and calculated transition-specific hazard ratios of LEI using Cox regression. The transition probabilities as well as the YLL due to LEI were estimated.

**Results:**

LEI significantly increased the risk for each transition, with a maximum risk for the transition from *Healthy* to *Care* (HR: 1.70, 95 % CI: 1.63-1.77) and a minimum risk for the transition from *Care* to *Dead* (HR: 1.16, 95 % CI: 1.10-1.22). If the elderly had LEI-history, their age-specific mortality was generally higher and their probabilities of transient states peaked at younger ages. At age 75, initially dementia-free and care-independent elderly experiencing LEI lost about 2 years of life, of which more than 90 % were life years free of dementia or care need. Dementia patients lost about one and a half year, more than 60 % were free of long-term care need.

**Conclusions:**

LEI not only casts a large health burden on care need, but is also associated with cognitive decline and shortened rLE. LEI plus dementia extend the relative life time in need of care, despite generally shortening rLE. Using the composite measure YLL may help to better convey these results to the elderly, families, and health professionals. This may strengthen preventive measures as well as improve timely and rehabilitative treatment of LEI, not only in cognitive and physically intact elderly.

**Electronic supplementary material:**

The online version of this article (doi:10.1186/s12877-016-0184-7) contains supplementary material, which is available to authorized users.

## Background

As the world’s population ages, age-dependent disorders are of great concern. Dementia, a key age-dependent disorder, dramatically contributes to disability and dependency, thereby challenging the health care system substantially [1].

Geriatric trauma, another age-related disorder, is also drawing more attention in public health [2]. With age, physiological reserve capability declines, physiologic reaction to injury degenerates, and age-related multimorbidity and polypharmacy worsen disability and mortality after injuries [3, 4]. Traumatic injuries also pose a challenge to health care [2], and rank as the fifth leading causes of death in elderly [3].

The lower extremities, covering the hip, thigh, knee, lower leg, ankle and foot, are one of the most likely region of the body for traumatic injuries in old adults [5, 6]. We focus on lower extremity injury (LEI), which includes not only hip fractures but also other types of fractures and fall-induced LEI, all of which increase the risk of mortality and deleterious disability and long-term care [7, 8]. LEI is also related with limitation of mobility and social participation [9, 10].

Numerous studies demonstrate an association between geriatric LEI and care need [7, 10–13]. LEI dramatically contributes to functional decline and increased dependency for basic and instrumental activities of daily living (ADL) [7, 10, 11]. In particular, dementia patients suffering the LEI are more likely to get poor functional outcomes and long-term care need [10, 14]. In turn, those living in long-term care institutions have also substantially increased rates of falls and fractures [12].

Previous research suggests an association between geriatric LEI and dementia [15–17]. Dementia increases the risk of falling and LEI disproportionately [18, 19]. In turn, LEI might lead to depression and delirium, as well as the restricted physical and social activity, which contributes to a cognitive decline and dementia onset [16, 20–24].

A bunch of studies reveal that geriatric LEI increases mortality considerably [8, 10, 25–29]. The mortality following LEI is even higher in people with dementia than people without dementia [25, 30, 31]. Moreover, the impairment in basic ADL (BADL) prior to LEI is also significantly associated with increased risk of mortality following LEI [32].

But most of the previous studies analyzed the associations separately. It lacks research to evaluate the effects of LEI on functional and survival outcomes during the aging process holistically. The aging process covers two major functional declines, namely physical decline and cognitive decline [33]. Dementia can be regarded as a later state of cognitive impairment [33], whereas the impairment in the BADL and the consequent care need can be regarded as a later state of physical impairment.

A holistic analysis of LEI effects in the aging is valuable because all forms of dementia, care need and premature mortality add a health burden on a society with an aging population, and also because LEI and dementia as well as mobility and cognitive dysfunction may interact and in particular aggravate one another, leading to a profound combined effect on care need and mortality [33, 34]. Moreover, the investigation of both survival and functioning outcomes simultaneously has also been recommended in the geriatric research such as LEI and successful aging [35, 36].

Hence, we used a multi-state model to simultaneously evaluate the effect of LEI on the endpoints dementia, care need, and death with the focus on the transitions from one state to another over age [37–39]. As successfully applied in previous studies, multi-state models help quantify how risk factors at the individual level alter life expectancy at the population level [40]. The compound measures derived from multi-state models, such as remaining life expectancy (rLE) and years of life lost (YLL), are highly intuitive to the public and to health professionals and help quantify the health burden and make intervention choices [40].

Additional methodological shortcomings in prior research include sample size and short-term follow up in longitudinal studies [26, 27]. Previous studies have commonly been based only in hospital settings, or used cohorts consisting only of voluntary participants, both of which excluded nursing home residents.

Therefore, we aimed to use a large nationally representative sample of Germans living in private households and in nursing homes with a 6-year follow-up to: (1) simultaneously investigate the association between LEI and dementia, care need, as well as death; (2) explore the transition probabilities of getting dementia, care need, and dying from various initial states, stratified by LEI; (3) estimate to what extent LEI influences rLE, as well as rLE with dementia and care need; and (4) quantify the consequences of LEI in terms of YLL.

## Methods

### Sample and study design

We used claims data from the largest public health insurer in Germany (AOK), which covered about one-third of the German population. A national 2.2 % random sample of AOK beneficiaries aged 50 years and older, regardless of whether they went to the doctor, was drawn in the first quarter of 2004. We used the data in 2004 to classify the initial states, and followed the 62,103 individuals in our sample who were between 75 and 95 years of age from 2005 to 2010. In this age range, usually various transitions to dementia, care need and death occur. Medical and care need data were available from both the inpatient and the outpatient sectors for each person for each quarter, except that the data of care need from 2004 to 2006 were documented only once a year. The dataset supporting the conclusions of this article is not publicly available. Data access was legally approved by the “Wissenschaftliches Institut der Ortskrankenkassen” (WIdO). The study is based on anonymised administrative claims data that never involved patients directly. Individual patients cannot be identified and the analyses presented do not affect patients whose anonymized records were used.

### Variables of interest

We used ICD-10 to identify dementia (Dementia: G30, G31.0, G31.82, G23.1, F00, F01, F02, F03, and F05.1). To account for false positive diagnoses we developed a validation procedure. First, only diagnoses indicated as “verified” by a medical doctor were included from outpatient services, while from inpatient services only the discharge and secondary diagnoses were considered. Second, only those diagnoses with a second occurrence in the same quarter by different types of physicians or over time were considered. The only exception was when a patient died immediately after a dementia diagnosis in the same quarter; all of these cases were considered valid dementia cases [41].

We defined care need as receiving benefits from the statutory long-term care insurance in Germany. Such insurance includes cover for long-term care at home or in an institution, and is statutory and compulsory for all citizens in Germany [42]. The long-term care benefits are available for all insured persons, irrespective of age or wealth. The Medical Advisory Service of the Statutory Health Insurance Funds assesses whether there is need of care. Based on the German law regarding long-term care insurance in the study period (2004-2010), care need in our study refers to a minimum of assistance for at least 90 min per day, with more than 45 min per day attributable to basic care in the BADLs such as washing, eating or dressing. This implies that dementia patients with intact ADLs do not receive benefits and, thus, in our study are not coded as being in need of care.

Our exposure of interest was LEI, namely injuries to the hip and thigh, the knee and lower leg as well as the ankle and foot [6]. We used ICD to identify LEI (S70 - S99 and its related T section of the ICD codes, see details in Additional file 1: Table S1).

### Model

**Fig. 1:**
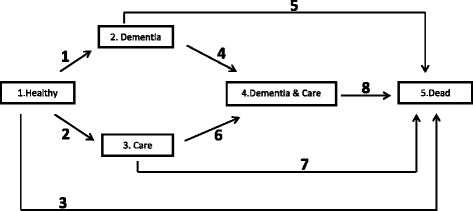
Multi-state model of the stages of healthy, dementia, care and dead. The boxes represent five states: (1) *Healthy* refers to no dementia, no care need. (2) *Dementia* includes incident and prevalent dementia, but without care need. (3) *Care* includes incident and prevalent care need, but without dementia (4) *Dementia & Care* refers to both dementia and care need. (5) *Dead* refers to a dead state, the only absorbing stage in our model. The other four states are transient states. The arrows represent the eight transitions from the ORIGIN STATE to the DESTINATION STATE

We applied a multi-state model to assess the risk of LEI for the eight possible transitions between the four transient states *Healthy*, *Dementia*, *Care*, *Dementia & Care,* and the only absorbing state *Dead* (Fig. 1). The state *Healthy* contains all individuals without a dementia diagnosis and without care need. Those with an incident or prevalent dementia diagnosis but without care need are contained in the state *Dementia*; those with incident or prevalent care need but without a dementia diagnosis are in the state *Care*. Insurants who have both a dementia diagnosis and are in need of care are included in the state *Dementia & Care*. The model does not consider recovery from dementia and from care need, which is reflected in Fig. 1 by the absence of the respective transitions. At present dementia cannot be treated and mild cognitive impairment, which can revert, has a separate ICD-10 number and is not part of our study. Recovery from care need is excluded because of the very small number of cases with a transition from care need to no care need. Individual persons may experience multiple transitions across different states during the follow-up period; as recovery is not possible, there are no multiple transitions of the same type.

We estimated stratified Cox proportional hazard models, with LEI as a time-dependent and transition-specific explanatory variable, sex as a stratification factor, and age as process time, under consideration of right censoring and left truncation [43]. Right censoring was identified as changing to another health insurer, reaching age 95 during the follow-up, and staying in one of the transient states on December 31, 2010. For each individual the date of birth was given; the date of death was recorded if he/she died during the study time; the date of the dementia and LEI diagnoses were also given by the quarter of the year which also applied to the variable care need between 2007 and 2010. Age on January 1, 2005 was used for the left truncation. In addition, when estimating transition hazards, for each transition, age of entry into the origin state of that transition was used as left truncation. We treated dementia, care need, and LEI as “ever”-variables, which have the value one the first time they are recorded in the data and thereafter; otherwise they have the value zero. Since care need before 2007 was only recorded annually, it was assumed to occur in the middle of the year if the individual survived to the end of that year; for individuals who died in that year it was placed in the middle of the survival period. In addition, given our quarter-based data, we assumed that all the transitions and LEI occurred in the midpoint of each quarter with the following exceptions: if an individual experienced multiple transitions and/or LEI in the same quarter of the year, then dementia, care need and LEI occurred before death; dementia occurred before care need; LEI occurred before care need and dementia. We conducted sensitivity analyses under different assumptions of the ordering which did not affect our results. We also checked the proportional hazard assumptions in the Cox models which were generally fulfilled.

We obtained the hazard ratio and 95 % confidence interval of LEI for each transition hazard in our multi-state model. The transition probability was defined as P_hj_(s,t) = P(X(t) = j|X(s) = h), which denoted the transition probability from state h to state j in the time (age) interval (s,t] [44], namely the probability that the subject is in state j at age t, given that he/she is in state h at age s. Estimates of the transition probabilities were obtained from the estimated transition hazards using the Aalen-Johansen formula [43, 45]. We estimated and plotted the age-specific transition probabilities of men and women by LEI in the age range between 75 and 95, starting at an individual’s 75^th^ birthday with various initial states. Furthermore, we estimated the state-specific rLE for a 75-year-old individual by calculating the state-specific expected duration of stay for a 75-year-old individual with various initial states. Moreover, we compared the state-specific rLE under LEI exposure and under no-LEI exposure by using the composite measure YLL due to LEI. We restricted the state-specific rLE from age 75 to 95 according to the age range of our study population. We bootstrapped 95 % confidence intervals for the state-specific rLEI and YLL by performing a thousand replications to resample the sample data with replacement. We used the “mstate” package in R 3.0 to perform the multi-state analysis [44].

## Results

In the first quarter of 2005 our sample comprised 62,103 insured persons (mean age ± standard deviation: 81.5 ± 4.8 years; 71.2 % female); of these 74 % (*N* = 45,758) were in the state *Healthy*, 5 % (*N* = 2,888) in the state *Dementia*, 13 % (*N* = 7,835) in the state *Care*, and 9 % (*N* = 5,622) in the state *Dementia & Care*. During the follow up, 25,730 persons died, 1,538 persons changed to other health insurance companies, and 6,651 persons reached ages over 95.

The first part of Table 1 (Col I-VI) shows the number of persons at risk of transitioning in each ORIGIN STATE (Col I) as well as the number of transitions from ORIGIN STATES to DESTINITION STATES during follow up (Col II to VI). Between 2005 and 2010, 45,758 individuals were at risk of transitioning from the state *Healthy* (Col I). Of these, 7,699 (17 %) experienced a transition to the state *Dementia*, 12,302 (27 %) to *Care*, 5,446 (12 %) to *Dead*, and 20,311 (44 %) remained in state *Healthy* through the end of the study or the time point of right censoring (Col II ~ VI). Of the 10,587 individuals in the risk population of the state *Dementia* between 2005 and 2010 (Col I), 6,977 individuals changed to the state *Dementia & Care* (Col IV), 1,349 to the state *Dead* (Col V), and 2,261 had no change (Col VI). The second part of Table 1 shows the number of persons with LEI and the proportion of LEI in each ORIGIN STATE (Col VII ~ VIII). 10,690 individuals had LEI-history in the state *Healthy* (Table 1, Col VII). In the states *Dementia* and *Care* about 30 % individuals had LEI-history (30.5 % and 31.9 % respectively), in the state *Dementia & Care* it was 41.7 % (Col VIII). Additional file 2: Table S2 details this information by 5-year age groups.

**Table 1:** Numbers and percentages of transitions and exposure of interest (LEI)

	*I*	*II*	*III*	*IV*	*V*	*VI*	*VII*	*VIII*
Origin State	Risk set (persons)^a^	Destination state				No change^b^	N of	% of LEI^c^
		2. Dementia	3. Care	4. Dementia & care	5. Dead		LEI	
1. Healthy	45,758	7699	12,302		5446	20,311	10,690	23.4
	100 %	17 %	27 %		12 %	44 %		
2. Dementia	10,587			6977	1349	2261	3228	30.5
	100 %			66 %	13 %	21 %		
3. Care	20,137			5734	7705	6698	6422	31.9
	100 %			28 %	38 %	33 %		
4. Dementia & Care	18,333				11,230	7103	7637	41.7
	100 %				61 %	39 %		

^a^numbers of individuals who were under the risk of the transitions from “ORIGIN STATE” to “DESTINATION STATE” on January 01,2005 or during the follow up. ^b^ numbers and percentage of individuals who entered or began with the particular ORIGIN STATE and stayed in that state until the end of the study or until the time point of right censoring. ^c^ proportion with LEI in the ORIGIN STATE

LEI accelerated health deterioration and significantly increased the risk of each transition (Table 2). LEI increased the risk of almost all transitions from the states *Healthy* or *Dementia* by about 50 % or more, whereas LEI increased the risk of all other transitions to a smaller extent (by 16–28 %). Among all eight transitions the effect of LEI was largest for the risk of a transition from *Healthy* to *Care*: it was 70 % higher for those with LEI than for those without LEI (HR: 1.70, 95%CI: 1.63-1.77). The two sexes did not differ significantly in their effect sizes (results not shown).

**Table 2:** Hazard ratio of LEI for each transition

Transition		Hazard ratio	95 % CI of HR	*P*
1	1 Healthy - > 2 Dementia	1.54	1.46–1.62	<0.001
2	1 Healthy - > 3 Care	1.70	1.63–1.77	<0.001
3	1 Healthy - > 5 Dead	1.24	1.16–1.33	<0.001
4	2 Dementia - > 4 Dementia & Care	1.46	1.39–1.54	<0.001
5	2 Dementia - > 5 Dead	1.50	1.33–1.69	<0.001
6	3 Care - > 4 Dementia & Care	1.26	1.19–1.33	<0.001
7	3 Care - > 5 Dead	1.16	1.10–1.22	<0.001
8	4 Dementia & Care - > 5 Dead	1.28	1.23–1.33	<0.001

**Fig. 2:**
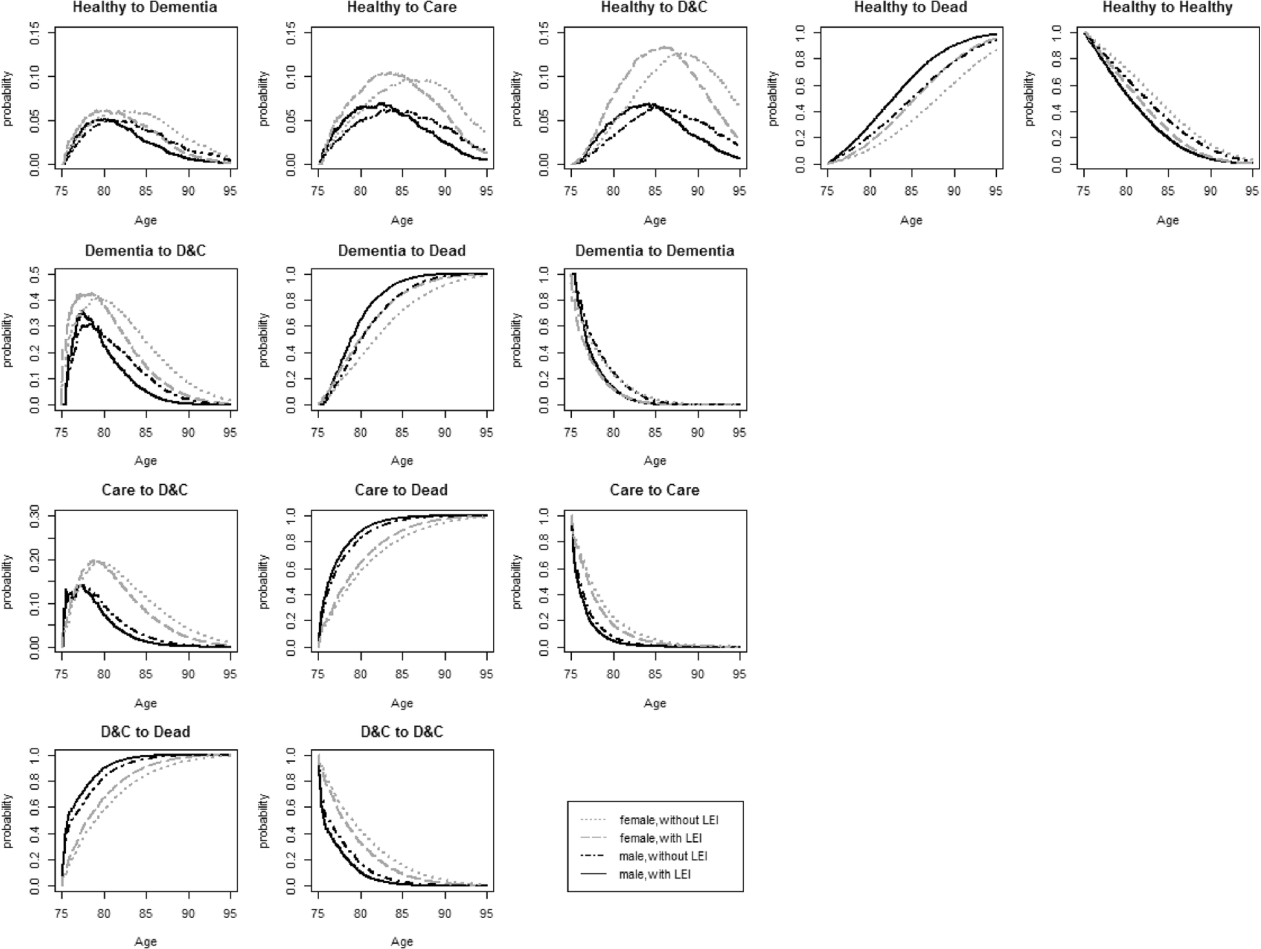
Estimated transition probabilities in the age interval 75–95, stratified by sex and LEI. For the populations with different initial states on the 75^th^ birthday. (1^st^ row: from *Healthy* as the initial state; 2^nd^ row: from *Dementia* as the initial state; 3^rd^ row: from *Care* as the initial state; 4^th^ row: from *Dementia & Care* as the initial state). ^”^Without LEI” refers to individuals who do not experience LEI during the whole period. “With LEI” refers to individuals who start out with LEI in their initial state. Abbreviation: D&C: *Dementia & Care*

Figure 2 shows the age-specific estimated probabilities of the transitions from the four initial states *Healthy* (1^st^ row), *Dementia* (2^nd^ row), *Care* (3^rd^ row) and *Dementia & Care* (4^th^ row), stratified by the presence of LEI and sex. For example, Fig. 2, 1^st^ row illustrates the transition probabilities for a synthetic cohort initiating in the state *Healthy* on the 75th birthday. At exact age 85, the probability of a woman without LEI to be in state *Dementia* was 5.7 %, in state *Care* 9.5 %, in state *Dementia & Care* 10.6 %, in state *Dead* 32.7 %, and to remain in the state *Healthy* 41.4 %. For most transitions, the age-specific probability for both sexes of entering a deteriorating state was larger for those with LEI than for those without LEI. Regarding the transitions to transient states, for both sexes the transition probabilities peaked at younger ages for those with LEI than for those without LEI. In the transitions with care need as the DESTINATION STATE (from *Healthy* to *Care*, or from *Dementia* to *Dementia & Care*), women had a markedly larger age-specific probability to experience these transitions than men. Regarding the transitions to the absorbing state death, men with LEI had the largest age-specific probability to die. In both sexes individuals with LEI always had a larger age-specific probability of dying than individuals without LEI.

For both sexes, the elderly with LEI spent generally shorter periods in each transient state than those without LEI (Table 3). At age 75, women who started healthy (initial state *Healthy*) but experienced LEI lost 2.10 years of rLE, males lost 1.97 years. More than 90 % of the years lost were years free of dementia and independent of long-term care (women 2.00 years, males 1.79 years), while the years with dementia and care need were reduced only marginally. Women who started with dementia (initial state *Dementia*) lost 1.72 years of rLE, men lost 1.35 years. Again, the majority of YLL were years with dementia but free of care need (women: 1.05 years = 61 %; men: 0.93 years = 69 %). The same was true for those who started with care need but were free of dementia. The YLL (women: 0.56 years = 68 %; men 0.33 years = 69 %) were mainly years with physical limitations but intact cognitive functioning. For most transitions the YLL due to LEI were significantly different from zero at conventional significance levels. Furthermore, the elderly with LEI experienced more years with dementia and care as compared to years with dementia alone. Taking a 75-year-old *healthy* woman as an example, under the exposure of no-LEI her rLE in the state *Dementia & Care* was 1.56 years, and 0.77 in the state *Dementia* which is a ratio of 2.03. For her counterpart with LEI, in contrast, the ratio is 2.53 (rLE 1.62 years *Dementia & Care*: rLE 0.64 years *Dementia*). A similar ratio existed for men, and for *Dementia* as the initial state.

**Table 3:** Remaining life expectancy at age 75 (rLE) and 95 % confidence intervals by initial state, stratified by sex and LEI

Initial state	Destination state	rLE without LEI^a^	rLE with LEI^a^	YLL due to LEI^b^
Females				
1. Healthy	1. Healthy	8.95	6.95	2.00
		(8.83 9.06)	(6.79 7.13)	(1.84 2.13)
	2. Dementia	0.77	0.64	0.13
		(0.74 0.81)	(0.59 0.68)	(0.09 0.18)
	3. Care	1.33	1.30	0.03
		(1.29 1.38)	(1.24 1.37)	(-0.03 0.09)
	4. Dementia & Care	1.56	1.62	−0.06
		(1.52 1.61)	(1.56 1.70)	(-0.13 0.01)
	Total	12.61	10.51	2.10
2. Dementia	2. Dementia	3.19	2.14	1.05
		(1.95 4.01)	(0.82 2.98)	(0.90 1.23)
	4. Dementia & Care	4.31	3.64	0.67
		(3.97 4.59)	(3.20 3.94)	(0.49 0.90)
	Total	7.50	5.78	1.72
3. Care	3. Care	3.40	2.84	0.56
		(2.86 3.89)	(2.31 3.33)	(0.43 0.69)
	4. Dementia & Care	1.96	1.70	0.26
		(1.63 2.38)	(1.38 2.12)	(0.16 0.37)
	Total	5.36	4.54	0.82
4. Dementia & Care	4. Dementia & Care	5.24	4.21	1.03
		(4.23 5.99)	(3.15 4.99)	(0.86 1.20)
Males				
1. Healthy	1. Healthy	7.96	6.17	1.79
		(7.81 8.12)	(5.96 6.38)	(1.63 1.94)
	2. Dementia	0.57	0.46	0.11
		(0.53 0.60)	(0.42 0.51)	(0.07 0.14)
	3. Care	0.82	0.79	0.03
		(0.79 0.87)	(0.74 0.85)	(-0.01 0.08)
	4. Dementia & Care	0.79	0.75	0.04
		(0.75 0.83)	(0.70 0.81)	(-0.01 0.07)
	Total	10.14	8.17	1.97
2. Dementia	2. Dementia	3.44	2.51	0.93
		(3.01 3.92)	(2.08 2.95)	(0.79 1.07)
	4. Dementia & Care	2.42	2.00	0.42
		(2.20 2.65)	(1.77 2.24)	(0.31 0.52)
	Total	5.86	4.51	1.35
3. Care	3. Care	1.74	1.41	0.33
		(1.30 2.18)	(0.99 1.82)	(0.24 0.42)
	4. Dementia & Care	0.91	0.76	0.15
		(0.61 1.25)	(0.48 1.07)	(0.09 0.24)
	Total	2.65	2.17	0.48
4. Dementia & Care	4. Dementia & Care	2.52	1.86	0.66
		(1.72 3.34)	(1.13 2.62)	(0.86 1.20)

*rLE* remaining life expectancy at age 75, *CI* 95 % Confidence intervals bootstrapped by 1000 replications,*YLL* years of life lost, ^a”^Without LEI” refers to individuals who do not experience LEI during the entire period. “With LEI” refers to individuals who start out with LEI at age 75 in their initial state. ^b^YLL due to LEI, calculated by the difference between the columns “rLE Without LEI” and “rLE With LEI”

## Discussion

Exploring individual transitions in a large population-based data set, we found that LEI in the elderly significantly increased the risk of the entire adverse chain from health over dementia and care need to death and resulted in a large loss of years of life. Similarly important, we found that these lost years were mainly years with better health and fewer limitations, independently of whether the individual was initially healthy or suffered from dementia or was in need of care. LEI increased all age-specific death probabilities (particularly in men), and shifted the age-peak of the probabilities of the transient transitions forward (particularly the transitions to care need in women). LEI was generally associated with shortened life expectancy, but with relatively expanded life time with dementia and care need.

To our knowledge, this is the first study to explore the effect of LEI on the risk of dementia, care need, and death simultaneously in a multi-state model. Earlier studies primarily looked at separate endpoints at the individual level and did not derive compound measures at the population level, such as life expectancy and years of life lost. Our findings provide insight into the effects of LEI on both the functional outcomes (dementia, care need) and the survival outcomes holistically, which is highly recommended in the research fields of successful aging [35].

Furthermore, we studied LEI instead of only hip fractures or fall-induced fractures, because they are not as fatal but are still associated with worse disability outcomes and long-term care need [7, 28] and result in enormous societal costs [1]. In addition, given the large number of underreported falls [46], LEI is easier to notice for a doctor, a caregiver or a family member.

Our results not only confirm findings in previous studies, but also deepen the knowledge about the association between LEI and dementia, care need and death. We shall now discuss them briefly.

### LEI and care need

We show that LEI increases the risk of care need, both among *healthy* elderly and among *dementia* patients. It is well known that fracture or fall-induced injuries in elderly are associated with poor functional outcomes and high burden of care, including longer stay in hospital and higher likelihood of long-term care facility [7, 13]. The presence of dementia aggravates these negative effects [47]. The underlying mechanisms might be that LEI speeds up the course of dementia [48], or that dementia slows down functional recovery after LEI, thus increasing care need in patients with dementia [34, 49].

In our study, women generally have a higher probability of experiencing the transition to long-term care than men, despite their higher rLE, thus, supporting the outcome of earlier studies [50, 51]; LEI shifts their age at the transition even further forward.

### LEI and dementia

We show that LEI is a risk factor or a predictor of dementia for both healthy individuals and for those with care need. Various mechanisms may explain the association between LEI and dementia. First, LEI in the elderly increases the risk of cognitive impairment, including delirium and depression [16, 20], which raises the risk of dementia onset [21, 52–54], and makes dementia worse and more progressive [55, 56]. Second, LEI restricts physical activity, mobility and social participation, at least during some periods of time [9, 10], thus restricting these potential preventive factors of dementia [22, 23]. The decreased mobility in long-term care residents, in particular, is associated with various psychosocial impairments such as depression and feeling of isolation [57]. Also, falls and the consequent fear of falling might lead to activity restriction in the long run [58, 59]. Third, there exist shared risk factors for LEI and dementia such as age, ApoE4, diabetes and vascular dysfunction, executive dysfunction and gait disturbances [15, 33].

Yet the observed association must be interpreted with caution because of the possibility of reversed causality. Although we have already taken temporality into account by only using LEI which occurred prior to or simultaneous with transitions as the exposure of interest, LEI can be regarded as a pre-symptom or an early symptom of undiagnosed dementia. Patients experiencing LEI may have already suffered from mild cognitive impairment or undiagnosed moderate dementia. Particularly, gait and balance disorders are common in Non-Alzheimer’s Dementia [60]. Moreover, delirium and depression may be two of the key intermediate factors between dementia and LEI, but the causal association between these and dementia is still controversial [54].

We find that women have a higher probability of developing dementia once they are in need of care, while both sexes have similar probabilities when they are healthy. This might explain why community dwelling cohort studies have not usually found significant sex-differences in the age-specific incidence of dementia [61], while studies including the institutionalized population do find higher female dementia incidence [41].

### LEI and mortality

We find that LEI generally increased the risk of death, and particularly did so among dementia patients. Higher mortality has been observed in several previous studies on fractures or falls [8, 10, 25–28]. Yet the underlying mechanism is still unclear. Some studies suggested that merely the event of the fracture and post fracture conditions related to trauma, instead of pre-existing co-morbidities, is mainly responsible for the excess mortality [8, 62], whereas others claimed that the underlying health or comorbidities are linked to a large part of fracture-mortality association [63]. In line with our results, several previous studies have also pointed out that dementia is an independent risk factor or a predictor for mortality after fractures [10, 25, 26, 30].

Regardless of the initial state, men always had larger death probabilities, and LEI made this even worse by further increasing age-specific death probabilities and moving the age of death forward. Consistently, many studies on fractures or falls in elderly have reported that males are at an increased risk for death after LEI [8, 26–28].

### Strengths and limitations of our study

In addition to the abovementioned strengths about the composite measure (YLL) and the holistic analysis (multistate model), our study has several further strengths. First, we use a large population-based sample of community dwelling and institutionalized elderly, avoiding bias due to sample selection and granting high statistical power. Second, we include a 6-year follow-up period, which permits us to analyze multiple transitions between different states and to explore the long-term effect of LEI with high statistical power. Finally, in comparison to self-reported or interview data, claims data is relatively objective, and free of recall bias or interview bias.

Our study also has several limitations. First, the dementia in our cases was defined by a doctor’s ICD diagnosis, which is prone to imperfect sensitivity and specificity. While we used an internal validation procedure to minimize the probability of false positive dementia diagnoses, we cannot account for underreporting. Second, in our data dementia may have already existed ahead of LEI, and LEI simply brought the dementia to clinical attention. Therefore, we interpreted the association observed between LEI and dementia with caution, particularly taking reverse causality into account. More research is needed to understand the interactions between cognitive and mobility decline [33], and other underlying mechanisms of the observed association between LEI and dementia.

Third, to evaluate the long-term effect, we defined LEI as an ever-variable, although some LEI may be apparently or actually cured. However, LEI can influence a patient in the long-term, even after recovery. A relationship has been found linking fall and subsequently fear-related avoidance of activity [58]. Fourth, we discussed our results in comparison with quite a few previous literature on hip fractures or falls, which might be not directly comparable. However, the hip fracture (S72) in our data is the most frequent among the first 3-digits subgroups of the ICDs chosen. Fall-induced injuries in older adults account for the majority of geriatric injuries and their related hospital admissions [3, 64]. Fifth, we have no information about other potential confounders such as education, family status, or life style factors such as smoking, obesity, or alcohol consumption. Our multi-state model also does not control for polypharmacy or multimorbidity. However, in sensitivity analyses of our hazard models we further controlled for the Charlson-morbidity-index, brain injuries, osteoporosis, depression, Parkinson and Down-Syndrom at baseline, which did not alter our findings (not shown).

Moreover, given the crude nature of the quarterly-based data, we had to make assumption on the order in which LEI, dementia, care need and death occurred, in case more than one event happened within one quarter. Sensitivity analysis changing these orders still found a significantly increased risk of LEI for most transitions, albeit of smaller effect sizes (not shown).

## Conclusion

Our study suggests that prevention and timely treatment of LEI as well as rehabilitative care after LEI may be of utmost importance in delaying or reducing the onset of care need and death, not only among cognitive intact elderly but also among dementia patients. These measures will not only help to save years of life, but also to increase the quality of life of the elderly and reduce the burden for families, public health, and the care system. Expressing the detrimental effect of LEI in terms of the composite measure YLL should prove helpful for the elderly, their families, and health professionals to holistically appreciate the scale of the problem and the substantial benefits of preventive and rehabilitative measures.

## Additional files

Additional file 1: Table S1. ICDs used to identify dementia and lower extremity injury (ICD- 2010-GM). To list the concrete ICDs, which we used to identify dementia and lower extremity injury in our study. (DOCX 15 kb) Additional file 2: Table S2. Numbers and percentages of transitions and exposure of interest (LEI) by age. Differentiates Table 1 which is included in the main document by 5-year age groups. (DOCX 19 kb)

## Abbreviations

ADL: activities of daily living
BADL: basic activities of daily living
CI: confidence interval
Col: column
HR: hazard ratio
ICD: International Statistical Classification of Diseases and Related Health Problems
LEI: lower extremity injury
rLE: remaining life expectancy
SD: standard deviation
WIdO: Wissenschaftliches Institut der Ortskrankenkassen
YLL: years of life lost
