# Supplementary material for: Years of life lost due to lower extremity injury in association with dementia, and care need: a 6-year follow-up population-based study using a multi-state approach among German elderly
Source: BMC Geriatr. 2016 Jan 12;16:9. doi: 10.1186/s12877-016-0184-7 (PMC4710990; doi:10.1186/s12877-016-0184-7)
Supplement: Additional file 1: Table S1. — ICDs used to identify dementia and lower extremity injury (ICD- 2010-GM). To list the concrete ICDs, which we used to identify dementia and lower extremity injury in our study. (DOCX 15 kb) [file 12877_2016_184_MOESM1_ESM.docx]

**SUPPLEMENTARY**

**Table 1S. ICDs used to identify dementia and lower extremity injury (ICD- 2010-GM)**

| ***Dementia*** |  |
| --- | --- |
| G30 | Alzheimer disease |
| G31.0 | Circumscribed brain atrophy |
| G31.82 | Lewy body dementia |
| G23.1 | Progressive supranuclear ophthalmoplegia [Steele-Richardson-Olszewski] |
| F00 | Dementia in Alzheimer disease |
| F01 | Vascular dementia |
| F02 | Dementia in other diseases classified elsewhere |
| F03 | Unspecified dementia |
| F05.1 | Delirium superimposed on dementia |
| ***Lower Extremity Injury*** | |
| S70-S79 | Injuries to the hip and thigh |
| S80-S89 | Injuries to the knee and lower leg |
| S90-S99 | Injuries to the ankle and foot |
| T00.3 | Superficial injuries involving multiple regions of lower limb(s) |
| T00.6 | Superficial injuries involving multiple regions of upper limb(s) with lower limb(s) |
| T01.3 | Open wounds involving multiple regions of lower limb(s) |
| T01.6 | Open wounds involving multiple regions of upper limb(s) with lower limb(s) |
| T02.3 | Fractures involving multiple regions of one lower limb |
| T02.5 | Fractures involving multiple regions of both lower limbs |
| T02.6 | Fractures involving multiple regions of upper limb(s) with lower limb(s) |
| T03.3 | Dislocations, sprains and strains involving multiple regions of lower limb(s) |
| T03.4 | Dislocations, sprains and strains involving multiple regions of upper limb(s) with lower limb(s) |
| T04.3 | Crushing injuries involving multiple regions of lower limb(s) |
| T04.4 | Crushing injuries involving multiple regions of upper limb(s) with lower limb(s) |
| T05.3 | Traumatic amputation of both feet |
| T05.4 | Traumatic amputation of one foot and other leg [any level, except foot] |
| T05.5 | Traumatic amputation of both legs [any level] |
| T05.6 | Traumatic amputation of upper and lower limbs, any combination [any level] |
| T12 | Fracture of lower limb, level unspecified |
| T13 | Other injuries of lower limb, level unspecified |
| T24 | Burn and corrosion of hip and lower limb, except ankle and foot |
| T25 | Burn and corrosion of ankle and foot |
| T33.6 | Superficial frostbite of hip and thigh |
| T33.7 | Superficial frostbite of knee and lower leg |
| T33.8 | Superficial frostbite of ankle and foot |
| T34.6 | Frostbite with tissue necrosis of hip and thigh |
| T34.7 | Frostbite with tissue necrosis of knee and lower leg |
| T34.8 | Frostbite with tissue necrosis of ankle and foot |
| T35.5 | Unspecified frostbite of lower limb |
| T87.1 | Complications of reattached (part of) lower extremity |
