# Supplementary material for: Years of life lost due to lower extremity injury in association with dementia, and care need: a 6-year follow-up population-based study using a multi-state approach among German elderly
Source: BMC Geriatr. 2016 Jan 12;16:9. doi: 10.1186/s12877-016-0184-7 (PMC4710990; doi:10.1186/s12877-016-0184-7)
Supplement: Additional file 2: Table S2. — Numbers and percentages of transitions and exposure of interest (LEI) by age. Differentiates Table 1 which is included in the main document by 5-year age groups. (DOCX 19 kb) [file 12877_2016_184_MOESM2_ESM.docx]

**SUPPLEMENTARY**

**Table 2S. Numbers and percentages of transitions and exposure of interest (LEI) by age**

|  |  | ***I*** | ***II*** | ***III*** | ***IV*** | ***V*** | ***VI*** | ***VII*** | ***VIII*** |
| --- | --- | --- | --- | --- | --- | --- | --- | --- | --- |
| **ORIGIN STATE** | **AGE ^a^** | **Risk set** | **DESTINATION STATE** | | | | **No change^c^** | **N of** | **% of LEI^d^** |
|  |  | **(person)^b^**  **^(100%)^** | **2.Dementia** | **3.Care** | **4.Dementia& Care** | **5.Dead** |  | **LEI** |  |
| **1.Healthy** | **75 - <80** | 24383 | 3250 (13%) | 4916 (20%) |  | 2614 (11%) | 13603 (56%) | 5711 | 23.4 |
|  | **80 - <85** | 15055 | 3019 (20%) | 4800 (32%) |  | 1943 (13%) | 5293 (35%) | 3552 | 23.6 |
|  | **85 - <90** | 4379 | 1009 (23%) | 1909 (44%) |  | 657 (15%) | 804 (18%) | 1034 | 23.6 |
|  | **90 - <95** | 1941 | 421 (22%) | 677 (35%) |  | 232 (12%) | 611 (31%) | 393 | 20.2 |
| **2.Dementia** | **75 - <80** | 4211 |  |  | 2500 (59%) | 432 (10%) | 1279 (30%) | 1264 | 30.0 |
|  | **80 - <85** | 4121 |  |  | 2907 (71%) | 504 (12%) | 710 (17%) | 1270 | 30.8 |
|  | **85 - <90** | 1518 |  |  | 1137 (75%) | 264 (17%) | 117 (8%) | 475 | 31.3 |
|  | **90 - <95** | 737 |  |  | 433 (59%) | 149 (20%) | 155 (21%) | 219 | 29.7 |
| **3.Care** | **75 - <80** | 6754 |  |  | 1505 (22%) | 2637 (39%) | 2612 (39%) | 2159 | 32.0 |
|  | **80 - <85** | 7534 |  |  | 2202 (29%) | 2868 (38%) | 2464 (33%) | 2452 | 32.5 |
|  | **85 - <90** | 3596 |  |  | 1299 (36%) | 1470 (41%) | 827 (23%) | 1173 | 32.6 |
|  | **90 - <95** | 2253 |  |  | 728 (32%) | 730 (32%) | 795 (35%) | 638 | 28.3 |
| **4.Dementia & Care** | **75 - <80** | 5087 |  |  |  | 2714 (53%) | 2373 (47%) | 2118 | 41.6 |
|  | **80 - <85** | 6941 |  |  |  | 4317 (62%) | 2624 (38%) | 2949 | 42.5 |
|  | **85 - <90** | 3754 |  |  |  | 2762 (74%) | 992 (26%) | 1645 | 43.8 |
|  | **90 - <95** | 2551 |  |  |  | 1437 (56%) | 1114 (44%) | 925 | 36.3 |

^a^ age group according to the age on January 01,2005. ^b^numbers of individuals who were under the risk of the transitions from “ORIGIN STATE” to “DESTINATION STATE” on January 01,2005 or during the follow up. ^c^ numbers and percentage of individuals who entered or began with the particular ORIGIN STATE and stayed in that state until the end of the study or until the time point of right censoring. ^d^ proportion with LEI in the ORIGIN STATE.
